# The Oral and Nasal Gateway Microbiomes: Salivaomics and Systemic Health at the Airway–Digestive Interface

**DOI:** 10.3390/microorganisms14091913

**Published:** 2026-08-29

**Authors:** Mark L. Cannon, John Peldyak, Paul R. Reynolds, Gustavo Ferrer

**Affiliations:** 1 Ann and Robert H. Lurie Children’s Hospital of Chicago, Feinberg School of Medicine, Northwestern Universty, Chicago, IL 60611, USA; 2 Independent Researcher, Mount Pleasant, MI 48858, USA; 3 Department of Cell Biology and Physiology, Brigham Young University, Provo, UT 84602, USA; paul_reynolds@byu.edu; 4 Department of Pulmonary and Critical Care Medicine, Aventura Hospital and Medical Center, Aventura, FL 33180, USA; gferrer@moxie-health.com

**Keywords:** oral microbiome, nasal microbiome, oral–systemic health, salivaomics, periodontitis, oral–gut axis, oral–nasal airway axis, xylitol, obstructive sleep apnea, maternal–child health

## Abstract

The oral cavity is a mucosal and mineralized interface shared by the digestive tract and the upper airway. This narrative review proposes the oral–nasal gateway microbiome as a clinically useful model for understanding oral, nasal, and systemic health. The model includes bacteria, fungi, archaea, protozoa, viruses, bacteriophages, microbial metabolites, and host-derived salivary components. Its gateway role is supported by anatomy, continuous salivation, periodontal vascular exposure, oral–gut microbial overlap, nitrate–nitrite–nitric oxide biology, oral and nasal airway interactions, maternal–child microbial transmission, and enrichment of oral organisms in selected distal diseases and tumors. Oral communities respond rapidly to diet, salivary flow, airway physiology, smoking and vaping, xerostomic medications, antibiotics, and antiseptic rinses, and these changes may influence the nasal microbiome. Published evidence summarizes bacterial pathobionts and protective commensals; Candida and other oral fungi; herpesviruses; papillomaviruses; bacteriophages; salivaomics; pregnancy and early-life prevention; probiotics; polyols; remineralization chemistry; environmental exposures; and tumor microbiology. As of manuscript preparation, SalivaDB catalogs 15,821 salivary biomarker entries across 201 diseases and 48 disease categories. The practical endpoint is not sterilization of the oral cavity but restoration of microbial homeostasis, salivary competence, airway stability, dietary balance, and biologically informed, timely prevention.

## Simple Summary

The oral and nasal microbiomes can be understood as a gateway ecosystem because the oral cavity connects the upper digestive tract and the upper airway. Diet, saliva, oxygen tension, breathing route, host immunity, and microbial ecology meet at this interface. This narrative review examines bacteria, fungi, viruses, and bacteriophages as members of a co-evolved oral–nasal ecosystem that supports health when balanced and contributes to disease when disrupted. Evidence associates oral and nasal dysbiosis with caries, periodontal disease, cardiometabolic disease, adverse pregnancy outcomes, respiratory disease, allergies, and cancer-associated microbial ecology. Salivaomics offers a practical, noninvasive route for earlier biological risk detection in dentistry and respiratory medicine.

## 1. Introduction

The oral cavity contains one of the most diverse microbial ecosystems of the human body. It includes tooth-associated, gingival, tongue, palatal, buccal, tonsillar, saliva-associated, and oropharyngeal niches, each with distinct oxygen gradients, host surfaces, immune exposures, and nutrient flows [1,2,3,4,5,6,7]. Because the mouth is both the beginning of the digestive tract and a functional part of the upper airway, it should not be treated as an isolated dental compartment. It is a gateway ecosystem through which food, fluids, air, environmental particles, medications, host secretions, and microbes are sampled before they reach the gut, airways, circulation, or the maternal–fetal interface. The nasal gateway microbiome is understudied, yet it directly influences systemic health through mucociliary defense, nasal nitric oxide production, and continuous microbial crosstalk with the oral cavity. Whole-genome deep sequencing of the nasal microbiome has revealed a complex community dominated by Staphylococcus, Corynebacterium, Propionibacterium, and Dolosigranulum species in health, with striking shifts toward *Staphylococcus aureus*, Haemophilus, and Moraxella in disease states including allergic rhinitis, chronic rhinosinusitis, and obstructive sleep apnea [8]. The nasal and oral microbiomes share bacterial taxa, exchange organisms through the nasopharynx, and are subject to viral crosstalk, including respiratory viruses that colonize both compartments simultaneously and alter the downstream microbial ecology of each.

This review uses the term “oral gateway microbiome” as an evidence-based organizing model, not merely a metaphor. It describes a co-evolved barrier microbiome that contributes to colonization resistance, mucosal education, mineral homeostasis, nitric oxide biology, wound repair, and metabolic signaling. In health, the oral microbiome is dominated by cooperative microbial networks that restrain pathobionts and maintain host tolerance. In dysbiosis, ecological balance shifts toward acidogenic, proteolytic, invasive, or inflammatory organisms and toward host responses that fail to resolve tissue damage [9,10,11,12,13,14,15,16].

This organizing model is deliberately distinct from the oral and nasal gateway microbiome frameworks, and it is important to state explicitly what it adds. The oral–gut axis and oral–lung axis describe trafficking of oral organisms, metabolites, or inflammatory mediators toward a single distal organ system; neither, by itself, specifies a shared anatomic and physiologic checkpoint through which multiple exposure routes (inhaled, ingested, and swallowed) converge before reaching those organs. Oral–nasal microbial continuity documents that the two compartments exchange taxa across the nasopharynx, but continuity is a descriptive observation rather than a functional claim about barrier physiology or directionality. Mucosal microbiome models emphasize cross-site correlation and co-occurrence across many mucosal surfaces without specifying anatomic sequence or a rate-limiting interface. Oral–systemic medicine is the broadest of these frameworks and encompasses any mechanistic link between oral status and systemic disease, including pathways that do not pass through the nasal or airway compartment at all, such as direct hematogenous translocation from periodontal pockets. The gateway construct used here is more specific than any of these: it designates the combined oral and nasal mucosal surfaces as the anatomic checkpoint through which essentially all inhaled, ingested, and swallowed material—and the microorganisms it carries—must pass before reaching the gut, lower airway, systemic circulation, or maternal–fetal interface, and it treats the co-occurring, bidirectionally exchanging oral and nasal communities as a single functional unit rather than as two microbiomes connected by an axis. This checkpoint framing is supported by the anatomic continuity of the aerodigestive tract, direct microbial exchange demonstrated across the oral–nasal interface, and the shared dependence of both compartments on common host defenses, including mucociliary clearance, secretory immunoglobulin A, and antimicrobial peptides [17,18,19]. We use “gateway” rather than “axis” or “network” specifically to emphasize this checkpoint property; the term is not intended to replace the oral–gut axis, oral–lung axis, or oral–systemic medicine studies, each of which addresses a downstream consequence of passage through the gateway rather than the checkpoint itself. Its importance lies in the explicit anatomic and functional unification of the oral and nasal compartments as a single upstream checkpoint, not in the discovery of any single mechanistic link already described by these other frameworks.

Dental medicine has historically recognized the microbial nature of caries and periodontal disease, yet clinical systems often separate oral disease from general medicine. That separation is inconsistent with current evidence. Oral taxa overlap with the intestinal microbiome in a substantial subset of individuals; oral bacteria are continuously swallowed, and inflamed periodontal tissues permit microbial products and inflammatory mediators to reach systemic compartments [9,20,21,22,23,24]. Clinical associations with diabetes, atherosclerotic cardiovascular disease, adverse pregnancy outcomes, respiratory disease, inflammatory bowel disease, rheumatoid arthritis, neurodegenerative disease, and cancer vary in causal strength, but the evidence increasingly warrants integrating oral biology into systemic prevention [20,21,22,23,24,25,26,27,28]. Research into the nasal gateway microbiome has been sparse, and medicine has been more inclined toward surgical intervention than toward biofilm restoration. Recently published evidence now suggests that nasal dysbiosis may precede or amplify oral dysbiosis, and that pathobionts seeding the nasal passages can descend into the oropharynx and be swallowed, contributing to gastrointestinal colonization by organisms such as *Staphylococcus aureus*, *Haemophilus influenzae*, and *Streptococcus pneumoniae* [8,29].

The oral and nasal components of this framework are not supported by equivalent bodies of evidence. Oral microbiome research rests on a substantially larger, more mature, and more clinically validated literature than nasal microbiome research across most of the domains considered in this review. This asymmetry is acknowledged explicitly throughout: claims concerning the nasal gateway microbiome are presented, deliberately, in more hypothesis-oriented language than the corresponding oral microbiome claims, and the two should not be considered equally well established.

This review brings together evidence on oral bacteria, fungi, viruses, bacteriophages, salivaomics, maternal–child prevention, oral–airway biology, probiotics, polyols, environmental exposures, and tumor microbiology. The purpose is to present a clinically useful model that recognizes oral–systemic connections while avoiding single-pathogen explanations or unsupported claims of causality.

## 2. Scope and Narrative Search Strategy

This article is a narrative review. PubMed, PubMed Central, journal websites, and publisher databases were searched for research on the oral microbiome, oral–gut axis, oral–airway axis, periodontitis, dental caries, oral mycobiome, oral virome, bacteriophages, salivaomics, salivary biomarkers, maternal oral health, adverse pregnancy outcomes, probiotics, prebiotics, xylitol, polyols, microplastics, tumor microbiome, *Fusobacterium nucleatum*, and *Porphyromonas gingivalis*. We prioritized primary studies, systematic reviews, consensus statements, and recent mechanistic reviews. The goal was mechanistic and translational synthesis rather than quantitative pooling.

Searches covered January 2000 through March 2026, with emphasis on research published after 2010; no formal date limit was applied to foundational mechanistic or anatomic studies. Records were screened for relevance to the oral or nasal microbiome and human health outcomes; case reports without mechanistic or population context, non-peer-reviewed sources, and studies not available in English were excluded. Because this is a narrative rather than a systematic review, study selection was not exhaustive; this is acknowledged as a methodological limitation in Section 15.

## 3. Definition and Evolutionary Biology of the Oral and Nasal Gateway Microbiomes

### 3.1. A Co-Evolved Airway–Digestive Interface

The oral microbiome can be defined as the co-evolved microbial and viral ecosystem of the mouth and oropharynx, embedded in saliva and biofilm matrices, interacting with mineralized tooth surfaces, mucosal epithelium, gingival crevicular fluid, immune cells, dietary substrates, airflow, and swallowed secretions. This definition differs from narrower plaque-centered definitions by recognizing the oral cavity as a joint airway–digestive interface. The teeth and gingiva create non-shedding mineralized surfaces that permit the formation of mature biofilms, while the tongue dorsum, tonsillar region, palate, and saliva connect the oral cavity to the nasopharynx, oropharynx, esophagus, stomach, lungs, and gut [1,2,3,4,5,6,7,30].

The oral gateway microbiome is therefore specialized for intermittent nutrient pulses, mechanical shear, oxygen variation, pH cycling, host antimicrobial peptides, immunoglobulin A, nitrate-rich vegetables, salivary mucins, and repeated microbial immigration from food, water, air, hands, kissing, family contact, and dental procedures. This ecology explains why oral communities can change quickly when eating frequency, exposure to fermentable carbohydrates, breathing route, salivary flow, medications, or antimicrobial habits change [6,7,31,32,33,34,35,36].

### 3.2. Oral, Nasal, and Placental Gateway Microbiomes

The gateway microbiome concept extends beyond the mouth. The nasal microbiome is an airway gateway that conditions inhaled air, supports colonization resistance, maintains mucociliary and immune homeostasis, and interacts with nasal nitric oxide biology. Whole-genome deep sequencing has characterized the nasal microbiome, identifying *Staphylococcus epidermidis*, *Corynebacterium accolens*, *Propionibacterium acnes*, and *Dolosigranulum pigrum* as dominant commensals that contribute to competitive exclusion and mucosal immune education in healthy adults [8]. Disruption of this commensal community, documented in allergic rhinitis, asthma, and obstructive sleep apnea, is associated with enrichment of *Staphylococcus aureus*, *Haemophilus influenzae*, *Moraxella catarrhalis*, and *Streptococcus pneumoniae* [8,29]. The nasal and oral microbiomes are anatomically continuous through the nasopharynx and interact via shared airflow, microbial migration, and viral crosstalk; respiratory viruses that alter one compartment predictably reshape the other, establishing a bidirectional ecological vulnerability that has not been adequately incorporated into clinical practice [29]. The nasal microbiome also exerts systemic effects: dysbiosis in the nasal passages has been mechanistically linked to allergic sensitization, airway inflammation in asthma, and upper airway instability contributing to obstructive sleep apnea, in part through altered mucosal immune signaling and reduced nasal nitric oxide production [8,37]. The placental or maternal–fetal interface is a gateway in pregnancy through which microbial DNA, microbial metabolites, extracellular vesicles, inflammatory mediators, and, in pathologic settings, viable organisms may influence fetal immune priming and pregnancy outcomes [38,39,40,41,42,43,44]. The placental microbiome remains debated. Aagaard and colleagues reported a low-biomass placental microbiome that was most similar to the oral microbiome [38]. Later contamination-controlled studies argued that a healthy term placenta does not contain a typical resident microbiome [39]. These conclusions can be reconciled clinically by distinguishing a stable resident placental microbiota from a maternal–fetal microbial-signaling interface. The latter is supported by studies of oral-associated organisms in adverse pregnancy contexts, maternal microbial extracellular vesicles, immune activation, and microbiota-derived metabolites [40,41,42,43,44].

### 3.3. Responsiveness to Airway and Eating Patterns

A defining feature of the oral and nasal gateway microbiomes is their rapid responsiveness to airway and dietary conditions. Frequent consumption of sucrose, refined starch, acidic beverages, and ultra-processed foods selects for acidogenic and aciduric communities, including mutans streptococci and lactate-utilizing microflora that promote caries. Dietary nitrate from vegetables enriches nitrate-reducing taxa that support the nitrate–nitrite–nitric oxide pathway. Fasting, circadian timing, hydration, and salivary stimulation alter clearance and pH buffering [31,32,33,34,35,36,44,45,46,47,48,49].

Airway physiology is equally relevant. Nasal obstruction and mouth breathing dehydrate oral surfaces, alter oxygen exposure and salivary pellicle formation, and may favor the growth of inflammatory biofilms. Obstructive sleep apnea exposes oral and periodontal tissues to intermittent hypoxia, oxidative stress, sympathetic activation, and inflammatory signaling. Recent studies and reviews of obstructive sleep apnea, periodontitis, and oral microbiota support the view that airway state and oral dysbiosis interact rather than operate as separate clinical domains [50,51,52]. Table 1 lists representative components of the oral gateway microbiome.

## 4. Bacterial Members: Commensals, Pathobionts, and Ecological Function

Oral bacterial communities are spatially organized and metabolically interdependent. Early colonizers such as *Streptococcus sanguinis*, *Streptococcus mitis*, *Streptococcus gordonii*, *Actinomyces* spp., *Rothia* spp., and *Neisseria* spp. contribute to biofilm architecture and, in many contexts, are compatible with health. Veillonella spp. metabolize lactate and participate in cross-feeding. *Fusobacterium nucleatum* acts as a bridging organism that links early and late colonizers, contributes to coaggregation, and can become a systemic pathobiont when dysbiotic contexts permit invasion or distal enrichment [1,2,6,9,11,12,13,14,15].

*Streptococcus mutans* illustrates diet-responsive dysbiosis. Its acid production, acid tolerance, extracellular polysaccharide synthesis, and biofilm competitiveness increase with frequent exposure to fermentable carbohydrates. *S. mutans* is neither the only caries organism nor merely a harmless commensal; it is a disease-associated member of a broader acidogenic community. The caries model, therefore, supports an ecological rather than a single-pathogen interpretation [31,32,33,53,54,55,56].

Periodontitis provides the clearest example of polymicrobial dysbiosis. The red complex organisms *P. gingivalis*, *T. denticola*, and *T. forsythia* remain clinically relevant, but periodontal destruction is better explained by polymicrobial synergy and dysbiosis than by a single pathogen acting alone. *P. gingivalis* can modify complement and Toll-like receptor signaling, impair neutrophil control, and reshape the community despite low abundance. Orange-complex organisms such as *F. nucleatum*, *Prevotella intermedia*, and *Campylobacter rectus* help form transitional biofilm networks that support later anaerobic communities [11,12,13,14,15,16,53].

The oral gateway model also recognizes beneficial functions. Nitrate-reducing bacteria such as Neisseria and Rothia participate in oral nitrate-to-nitrite conversion, linking the mouth to nitric oxide biology and vascular function. Excessive daily antiseptic suppression can reduce nitrate reduction and may alter blood pressure responses, underscoring why a healthy oral microbiome cannot be equated with a sterile mouth [45,46,47,48,49].

## 5. Fungi and Cross-Kingdom Biofilms

The oral mycobiome is less abundant than the bacteriome but clinically relevant. *Candida albicans* can exist as a commensal yeast, but changes in host immunity, salivary flow, denture use, antibiotics, diabetes, inhaled corticosteroids, chemotherapy, and dietary substrates can promote hyphal growth, biofilm formation, and mucosal disease [57,58,59,60,61]. Fungal–bacterial interactions are central to this behavior. *C. albicans* adheres to oral streptococci, contributes to extracellular matrix structure, and can alter carbohydrate metabolism and biofilm architecture in severe early childhood caries and denture stomatitis [59,60,61].

The fungal component also matters for systemic health because candidiasis is a marker of immune and metabolic vulnerability, particularly in diabetes, xerostomia, immunosuppression, HIV, cancer therapy, and older adults. In oral squamous cell carcinoma and potentially malignant disorders, mycobiome shifts may reflect altered mucosal immunity, local metabolism, and changes in the epithelial barrier. Current evidence does not justify treating the oral mycobiome as a primary cause of systemic disease, but it supports including it in oral–systemic risk assessment [57,58,59,60,61].

## 6. Viruses and the Phageome

The oral virome includes eukaryotic viruses and bacteriophages. Eukaryotic viruses such as herpes simplex virus, cytomegalovirus, Epstein–Barr virus, and human papillomaviruses influence oral health through latency, reactivation, immune modulation, ulceration, and oncogenesis. Herpesviruses have been studied in periodontitis because they can modulate immune responses and may create conditions permissive for bacterial overgrowth. HPV is causally linked to a subset of oropharyngeal squamous cell carcinomas, connecting the oral–airway gateway to cancer prevention through vaccination and screening awareness [62,63,64,65,66,67,68].

Bacteriophages are not passive passengers. They shape bacterial abundance, mediate gene transfer and bacterial competition, interact with CRISPR-Cas systems, and influence biofilm stability. Oral phage communities appear person-specific and persistent, yet they also respond to disease states and environmental exposures. Their ability to carry auxiliary metabolic genes or antimicrobial resistance determinants places them at the center of microbial ecology, especially as precision phage therapy and microbiome editing move toward clinical translation [66,67,68].

A complete definition of the oral gateway microbiome must therefore include bacteria, fungi, viruses, and phages. A bacteria-only model misses cross-kingdom biofilm behavior, herpes viral immune effects, HPV-associated cancer, and phage-mediated microbial evolution.

## 7. Mechanistic Routes from Oral Gateway Dysbiosis to Systemic Biology

The oral–gut axis is a principal mechanism. Segata and colleagues showed that oral and stool communities overlap in many Human Microbiome Project subjects, supporting movement between oral and intestinal habitats [9]. Experimental oral administration of *P. gingivalis* in mice altered gut microbiota composition, downregulated tight junction genes, increased serum endotoxin levels, and resulted in detectable bacterial DNA in the liver before broader systemic inflammatory changes were observed [20]. These findings support a causal pathway by which oral pathobionts can alter the gut barrier and immune–metabolic physiology under experimental conditions. Table 2 summarizes the different evidence-supported links between the oral gateway microbiome and systemic health.

Hematogenous dissemination is another route. Periodontal inflammation increases epithelial ulceration within periodontal pockets and exposes vascular surfaces to microbial products. Transient bacteremia can occur after chewing, toothbrushing, periodontal procedures, and episodes of inflammation. The clinical implication is not that every bacteremia causes disease, but that repeated microbial and inflammatory exposures may contribute to cumulative systemic burden in susceptible hosts [21,22,23,24,25,26,27,28].

Host response biology is central. Periodontitis is not only an infection; it is a dysregulated host–microbe interaction. Neutrophil hyperactivity, complement dysregulation, inflammasome activity, IL-1β, IL-6, TNF-α, prostaglandin signaling, osteoclast activation, and impaired resolution all link local dysbiosis to systemic inflammatory tone [11,12,13,14,15,16,69,70,71,72].

## 8. Oral Disease Burden and Consequences of Ignoring the Gateway Role

Oral diseases are among the most common non-communicable diseases. Global analyses and WHO summaries estimate that oral diseases affect more than 3.5 billion people, with untreated dental caries, severe periodontal disease, tooth loss, and oral cancer contributing to pain, infection, missed school and work, nutritional impairment, social stigma, and healthcare costs [73,74,75]. This persistent burden reflects more than inadequate surgical intervention capacity. It reflects a failure to treat oral disease as a microbial, dietary, airway, salivary, behavioral, and systemic problem.

Ignoring the oral gateway microbiome has clinical costs. Caries is often treated as a localized enamel event rather than as a sign of dietary ecology, microbial acidification, salivary dysfunction, and family transmission. Periodontitis is often treated as a gum problem rather than as a chronic inflammatory disease that may complicate diabetes, vascular health, pregnancy, and aging. Xerostomia is often treated after damage occurs, despite its role as a gateway failure of lubrication, buffering, antimicrobial defense, and remineralization. This repair-dominant model delays prevention until microbial and inflammatory disease has become structurally visible.

A gateway model suggests that dental professionals should measure, explain, and manage oral microbial ecology with the same consideration they apply to blood pressure, glucose, and airway assessment. It also suggests that medical professionals consider oral infections and dysbiosis when evaluating chronic systemic disease.

## 9. Cardiometabolic, Vascular, Respiratory, Neurologic, Autoimmune, and Renal Links

### 9.1. Diabetes, Obesity, and Metabolic Disease

The association between periodontitis and diabetes is clinically established and bidirectional. Hyperglycemia impairs neutrophil function, collagen metabolism, vascular repair, and host response, while periodontal inflammation can worsen systemic inflammatory burden and glycemic control [24,25,76]. Mechanistic studies further connect oral pathobionts to insulin signaling, endotoxemia, and liver–gut metabolic pathways [20,76,77,78].

The oral microbiome may also reflect obesity and metabolic syndrome. Acidogenic taxa, lactate-producing organisms, periodontal inflammation, meal and snack frequency, and sleep-disordered breathing create overlapping ecologies. The evidence is not yet sufficient to prescribe a single oral microbiome therapy for obesity, but it is sufficient to consider oral inflammation and dietary exposure to fermentable carbohydrates as part of cardiometabolic risk reduction [20,24,25,78].

### 9.2. Cardiovascular and Endothelial Biology

Periodontal disease is associated with atherosclerotic cardiovascular disease in epidemiologic and mechanistic studies and consensus reports. Proposed pathways include bacteremia, endothelial activation, systemic inflammatory mediators, platelet activation, molecular mimicry, and shared risk factors such as smoking, diabetes, age, diet, and socioeconomic status [26,27,28,76,79]. The strongest clinical statement is that periodontitis should be treated as a modifiable inflammatory burden and a risk marker that merits interprofessional communication and cooperation.

The nitrate–nitrite–nitric oxide pathway provides a direct beneficial oral–systemic route. Nitrate-reducing oral bacteria convert dietary nitrate to nitrite, which can be further reduced to nitric oxide. Antibacterial mouthwash can reduce oral nitrite production and attenuate vascular effects of dietary nitrate or exercise, showing that beneficial oral bacteria have systemic physiologic relevance [45,46,47,48,49].

### 9.3. Respiratory Disease and the Oral–Nasal–Airway Axis

The oral–nasal airway is a continuum. Oral pathogens can seed respiratory infections through aspiration, particularly in frail, hospitalized, or institutionalized populations. Mouth breathing, allergic rhinitis, nasal obstruction, adenotonsillar hypertrophy, craniofacial restriction, orthodontic factors, and obstructive sleep apnea alter oral dryness, oxygen tension, and inflammatory tone. A review of oral–airway mitochondrial biology describes obstructive sleep apnea as a pathologic form of chronic intermittent hypoxia that can interact with periodontal inflammation, endothelial dysfunction, and salivary microbial changes [50,51,52,53]. The nasal microbiome is integral to this picture. Deep sequencing studies have shown that *Staphylococcus aureus* nasal dominance and loss of protective Corynebacterium and Dolosigranulum commensals are associated with atopic sensitization, asthma severity, and upper airway instability in obstructive sleep apnea, with mucosal immune dysregulation linking nasal dysbiosis to airway inflammation at both local and systemic levels [8,37]. When the nasal gateway microbiome fails, the oral cavity may become a secondary reservoir for pathogens and pathobionts that are continuously swallowed, contributing to gastrointestinal spread and potentially amplifying the systemic inflammatory burden already generated by periodontal disease [29]. These observations raise the hypothesis that combined evaluation of the nasal and oral microbiomes, rather than treatment of either compartment in isolation, could be informative in non-responding chronic disease. This remains a research question: no controlled studies have yet tested whether combined microbiome assessment changes diagnosis, clinical decision-making, or patient outcomes, and this should not be read as a clinical practice recommendation.

### 9.4. The Oropharyngeal Microbiome

The oropharynx is the anatomical and microbial crossroads where the oral, nasal, and laryngeal microbiomes converge. Its mucosal surfaces harbor a distinct community that shares taxa with the oral cavity above and the respiratory tract below, while also receiving continuous inocula from nasal drainage, swallowed saliva, and retrograde gastric contents. In health, the oropharyngeal microbiome is dominated by Streptococcus, Veillonella, Prevotella, Fusobacterium, Rothia, and Haemophilus species, with community composition shaped by tonsil architecture, lymphoid immune tissue, oxygen gradients, and mucus flow [19]. Dysbiosis of the oropharyngeal microbiome has been documented in recurrent tonsillitis, obstructive sleep apnea with tonsillar hypertrophy, and HPV-associated oropharyngeal squamous cell carcinoma, underscoring the clinical relevance of this anatomical gateway to both airway and oncologic disease [19,65]. The oropharynx acts as a microbial milieu: nasal pathogens descending from dysbiotic nasal passages meet oral pathobionts ascending from the gingival crevice, and the resulting community may be more pathogenic than either source population alone. Tonsillar biofilms in recurrent tonsillitis harbor multiple species in common with periodontal biofilms, and surgical removal of tonsils in children with sleep-disordered breathing normalizes oropharyngeal microbial ecology, supporting the view that the tonsils function as both a microbial reservoir and an immune surveillance organ whose balance is disrupted by chronic dysbiosis [19]. This shared ecology supports joint ENT and dental assessment in patients with recurrent pharyngitis, obstructive sleep apnea, or documented oral dysbiosis.

### 9.5. The Nasal Microbiome, Paranasal Sinuses, Nitric Oxide, and Cardiovascular Health

The paranasal sinuses are among the highest producers of nitric oxide in the human body. Nasal nitric oxide, derived primarily from the maxillary and ethmoid sinuses, is produced by inducible and constitutive nitric oxide synthase isoforms expressed in the sinonasal epithelium and is continuously released into the nasal airstream during nasal breathing [80]. This nasal nitric oxide reservoir serves multiple functions: it acts as a first-line antimicrobial defense against inhaled pathogens and viruses, participates in mucociliary clearance by stimulating ciliary beat frequency, and contributes to pulmonary vascular tone through pulmonary circulation during nasal breathing [80,81]. Nasal nitric oxide concentrations are markedly reduced in primary ciliary dyskinesia, allergic rhinitis, and chronic rhinosinusitis, conditions that are also associated with abnormal nasal microbiome composition, suggesting a bidirectional relationship between the microbial ecology of the paranasal sinuses and sinonasal nitric oxide production [80,82]. Just as oral nitrate-reducing bacteria support systemic nitric oxide bioavailability through the oral nitrate–nitrite–nitric oxide pathway [45,46,47,48,49], nasal and paranasal microbial communities may modulate nitric oxide availability at the airway interface, influencing not only local mucosal defense but also systemic vascular physiology. Disruption of this pathway through nasal dysbiosis, antibiotic suppression of nasal commensal populations, or chronic sinonasal inflammation may reduce nasal nitric oxide production and contribute, alongside disruption of the oral nitrate reduction pathway, to a cumulative deficit in systemic nitric oxide availability that is relevant to blood pressure regulation, endothelial function, and cardiovascular risk [45,80]. This nasal–cardiovascular axis parallels the oral–cardiovascular axis described in the nitric oxide literature and represents an underexplored dimension of airway–systemic health integration that merits collaborative investigation between cardiologists, otolaryngologists, and dental practitioners.

### 9.6. The Nasal Microbiome and Nasal and Nasopharyngeal Cancer

The relationship between the nasal and nasopharyngeal microbiome and upper airway malignancy is an emerging area of investigation that mirrors the better-characterized oral–colorectal cancer connection. Nasopharyngeal carcinoma, a tumor with a distinct geographic distribution and a strong association with Epstein–Barr virus, has been studied for nasal microbial signatures that may contribute to or reflect the tumor microenvironment. Recent sequencing studies of the nasal microbiome in patients with nasopharyngeal carcinoma have identified enrichment of *Fusobacterium nucleatum*, Prevotella, and anaerobic taxa in the tumor-adjacent nasal mucosa, consistent with the broader pattern of pathobiont enrichment observed in oropharyngeal-associated aerodigestive cancers [83]. Whether these organisms participate in oncogenesis, are passively enriched by the altered mucosal environment of early malignancy, or serve as biomarkers of immune dysregulation remains to be determined. The oral–nasal ecosystem shares multiple taxa with the oropharyngeal environment where HPV-associated squamous cell carcinoma arises; nasal viral carriage and nasal mucosal immune competence may influence local immune surveillance, which determines whether HPV infection progresses to malignant transformation in the oropharynx [19,65,83]. Nasal dysbiosis may reduce local innate immune defenses, impair mucociliary clearance of oncogenic viruses, and contribute to the chronic inflammatory milieu that promotes epithelial transformation. Nasal microbiome profiling may therefore have future value as a component of upper airway cancer screening, particularly in populations at elevated risk for nasopharyngeal carcinoma, chronic rhinosinusitis with mucosal remodeling, and HPV-associated oropharyngeal malignancy [83]. This remains a research area of investigation rather than an established clinical test, but it situates the nasal gateway microbiome within the tumor microbiome literature, which already recognizes oral taxa as participants in distal cancer ecology. One study reported a distinct gut microbiome signature in early-stage lung cancer [84]; the relevance of this finding to the oral–nasal gateway model remains uncertain.

### 9.7. Neuroinflammatory, Autoimmune, and Renal Domains

Oral–systemic associations with Alzheimer’s disease, rheumatoid arthritis, chronic kidney disease, and mental health are biologically plausible and supported by varying levels of epidemiologic and mechanistic evidence [85,86,87,88]. *P. gingivalis* has been studied for gingipains, citrullination pathways, and neuroinflammatory mechanisms; periodontal inflammation has been associated with rheumatoid arthritis and chronic kidney disease; and oral microbial shifts have been investigated in mental disorders. Oral dysbiosis should currently be viewed as a modifiable contributor or biomarker within multifactorial diseases, not as a solitary cause until significantly more research into the occurrence rate of oral systemic disease after treatment of oral dysbiosis has been published.

## 10. Maternal–Child Health and Intergenerational Oral–Systemic Prevention

Pregnancy is a practical window for oral gateway intervention. Maternal periodontal inflammation, caries risk, salivary mutans streptococci, dietary frequency, airway health, sleep, and access to care can influence maternal inflammation, the risk of adverse pregnancy outcomes, vertical microbial transmission, infant colonization, early childhood caries, and long-term health trajectories [38,39,40,41,42,43,44,89,90,91,92,93,94,95,96].

The maternal–fetal interface should be addressed with scientific precision. The existence of a stable resident placental microbiome in healthy pregnancy remains debated [38,39]. However, oral organisms and oral-associated bacterial signals are repeatedly implicated in adverse pregnancy contexts. *F. nucleatum* is a prominent example because oral strains have been linked to intrauterine infection, preterm birth, stillbirth, neonatal sepsis, and inflammatory pregnancy complications in case reports, mechanistic work, and reviews [40,41,42,43,44,90,91,92].

Maternal oral health also affects infant microbial acquisition. Maternal xylitol chewing gum trials from Finland and Japan showed delayed or reduced mutans streptococci acquisition by children and reductions in childhood caries. The PPaX cluster-randomized trial in Malawi evaluated xylitol chewing gum among more than 10,000 pregnant participants and reported reductions in preterm birth and low birth weight compared with standard prenatal care plus education [93,94,95]. These findings support investigating prenatal modulation of the oral microbiome as a public health strategy, particularly in settings with limited access to dental care.

The maternal–child research argues for a bundled prevention model: prenatal oral screening, caries and periodontal risk assessment, xylitol gum or other polyol strategies where appropriate, dietary counseling, saliva-sharing education, airway and sleep screening, probiotics or prebiotics when evidence and safety support use, infant dental home linkage, and integration with Medicaid, WIC, HRSA, obstetric, pediatric, and dental systems [89]. This model treats early oral disease as an intergenerational microbial and inflammatory risk marker rather than a minor pediatric inconvenience.

## 11. Tumor Microbiome, Oral Pathobionts, and Cancer Ecology

Cancer biology increasingly recognizes tumors as ecosystems containing malignant cells, immune cells, stromal cells, endothelial cells, extracellular matrix, metabolites, viruses, fungi, and bacteria. The tumor microbiome is tumor type-specific and often intracellular. Nejman and colleagues studied 1526 tumors and adjacent normal tissues across seven cancer types and reported distinct microbial compositions by tumor type, with intratumoral bacteria present within both cancer and immune cells [97]. This study demonstrates the presence of microbes in tumors and now leads us to investigate how tumor-specific microbial communities influence immunity, metabolism, drug response, and prognosis.

Bullman’s work on *F. nucleatum* provides a direct connection between the oral microbiome and tumor ecology. Bullman and colleagues showed that Fusobacterium and co-occurring microbes persisted in colorectal cancer metastases; in xenograft models, metronidazole reduced Fusobacterium load, cancer cell proliferation, and tumor growth [98]. Subsequent work identified a specific *F. nucleatum* subsp. animalis clade (Fna C2) enriched in the colorectal cancer niche, with genetic features consistent with gastrointestinal colonization and metabolic adaptation [99].

The cancer literature should not yet be reduced to a claim that oral pathogens cause cancer. A more conservative model suggests that oral pathobionts contribute to tumor-promoting conditions through adhesion, invasion, β-catenin signaling, immune evasion, autophagy, stemness, metabolite production, extracellular vesicles, treatment resistance, and inflammatory remodeling. *F. nucleatum*, *P. gingivalis*, and *S. mutans* have each been studied in cancer-associated contexts, particularly colorectal, oral, pancreatic, and aerodigestive cancers [97,98,99,100,101,102,103,104,105]. One methodological controversy within this literature should be noted explicitly: a high-profile report linking microbial signatures to multiple cancer types was retracted in 2024 after independent reanalysis identified data processing errors; a related follow-up analysis by the original authors has since defended the robustness of cancer microbiome signals under a broader range of methodological evaluation, but this remains an active and unresolved methodological dispute rather than settled evidence [104].

For this reason, cancer-related claims should currently be approached with caution. The presence of oral organisms in tumors or metastases does not necessarily prove that those organisms initiated cancer, but it does identify microbial ecology as one component of tissue-specific tumor biology and a plausible target for mechanistic research [97,98,99,100,101,102,103,104,105].

## 12. Salivaomics and Biologically Integrated Dentistry

Saliva is a diagnostic biofluid, not simply moisture. Whole saliva contains microbial DNA, host DNA, RNA, microRNA, circular RNA, long noncoding RNA, proteins, peptides, hormones, enzymes, antibodies, glycoproteins, metabolites, lipids, electrolytes, inflammatory mediators, cell-free DNA, circulating tumor DNA signals, extracellular vesicles, exosomal biomarkers, and viable and nonviable microorganisms [106,107,108,109,110,111,112,113,114,115].

The scale of salivaomics is now measurable. Early salivaomics work described a core salivary transcriptome of approximately 180 messenger RNAs and a core salivary proteome of 1166 proteins. SalivaDB, a curated database of human salivary biomarkers, contains 15,821 entries for 201 diseases and 48 disease categories, including 7729 unique salivary biomarkers as of manuscript preparation. Entries include 6067 proteins, 3987 metabolites, 2909 microbes, 2272 miRNAs, and 586 genes; 742 biomarkers are reported as exosome-derived (see Table 3) [106].

The diagnostic implications for dentistry are broad but heterogeneous in maturity. Salivary diagnostics for caries risk, periodontal disease activity, peri-implant risk, oral fungal overgrowth, and viral reactivation are the most analytically and clinically advanced applications. Saliva can also support the investigation of oral cancer risk, diabetes, metabolic syndrome, cardiovascular biomarkers, neurodegenerative biomarkers, inflammatory burden, and treatment response, but for these domains the supporting evidence is presently associative and exploratory rather than clinically validated, and near-term diagnostic deployment should not be inferred from biomarker discovery alone (see Section 15). Saliva also supports microbiome sequencing, metabolomics, proteomics, host response profiling, and computational pattern recognition. Used responsibly, salivaomics can move dental practice from delayed structural repair toward earlier, noninvasive, clinically applicable risk detection [106,107,108,109,110,111,112,113,114,115].

Implementation should be carefully considered. Saliva varies with time of day, fasting, hydration, flow rate, menstrual cycle, pregnancy status, medications, mouth breathing, recent brushing, dental treatment, smoking, exercise, and collection method. Tests should be analytically valid, clinically validated, interpretable, and linked to an action plan. Salivaomics will augment examination, radiography, periodontal charting, airway screening, and medical history rather than replace them. Because saliva reflects not only oral microbial composition but also drainage of nasal secretions, immune activation, metabolic state, and host response signaling, validated salivary panels should be investigated that may enable detection of airway microbiome shifts, including nasal dysbiosis patterns associated with allergic sensitization, asthma exacerbation, and OSA progression, before structural disease becomes clinically apparent [106,110].

## 13. Probiotics, Prebiotics, Polyols, and Microbiome Restoration

### 13.1. Probiotics and Prebiotics

Probiotics are live microorganisms that confer a health benefit when administered in adequate amounts. In oral health, candidate probiotics include *Lactobacillus reuteri*, *Lactobacillus rhamnosus*, *Lactobacillus plantarum*, *Lactobacillus paracasei*, *Bifidobacterium* spp., *Streptococcus salivarius* K12 or M18, and other strain-specific candidates [116]. Mechanisms include adhesion competition, bacteriocin production, pH modulation, immune regulation, support of the epithelial barrier, a reduction in volatile sulfur compounds, interference with pathogen coaggregation, and recovery of colonization resistance after disruption [117,118,119,120,121,122,123,124,125]. In nasal health, far less has been published on targeted nasal probiotic strategies, even though gut probiotics have been extensively studied, and the nasal microbiome is equally accessible and arguably more directly relevant to respiratory, allergic, and airway disease. The nasal gateway microbiome has remained more elusive because sampling is more complex, the community is of lower biomass, and clinical interest has historically favored pharmaceutical or surgical management of rhinosinusitis and allergic airway disease over microbiome restoration [126]. Whether the principles governing oral and gut probiotic restoration extend to the nasal passage remains an untested hypothesis rather than an evidence-based conclusion; mechanistic plausibility grounded in shared mucosal biology does not, by itself, establish the efficacy or safety of nasal probiotic strategies. Candidate nasal probiotics include *Lactobacillus rhamnosus*, *Lactobacillus casei*, and *Streptococcus salivarius*, which have demonstrated colonization resistance activity against nasal respiratory pathogens in early trials; administration via nasal spray or nebulization may offer a practical delivery route that bypasses gastrointestinal degradation [127]. The scarcity of nasal probiotic research relative to gut and oral research likely reflects the absence of an organized clinical specialty invested in nasal microbiome restoration, the absence of a diagnostic framework analogous to salivary microbiome profiling, and the lower commercial investment in non-antibiotic nasal therapeutics. These are structural gaps rather than biological barriers, and they must be resolved if the nasal gateway microbiome is to receive the same preventive and therapeutic attention as its oral counterpart. Oral probiotics may indirectly benefit the nasal passages through the gut–airway immune axis; probiotic supplementation in infancy and pregnancy has been associated with reductions in upper respiratory infections, allergic sensitization, and asthma risk, outcomes consistent with improvements in systemic mucosal immune programming rather than direct nasal colonization [127,128]. This systemic effect of probiotics on respiratory outcomes supports a multi-compartment model of microbiome restoration spanning dentistry, otolaryngology, pulmonology, and immunology.

The evidence for oral probiotics is promising but heterogeneous, partly because intervention protocols vary substantially. Most trials and meta-analyses show reductions in salivary *S. mutans*, gingival inflammation, bleeding indices, or periodontal pathogen burden, while others show modest or inconsistent clinical effects. Benefits depend on strain, dose, delivery vehicle, baseline disease state, diet, salivary flow, adherence, and whether the probiotic is used alone or as an adjunct to mechanical debridement and behavioral change [117,118,119,120,121,122,123,124,125]. The most significant inconsistency is the duration of probiotic use: trials reporting benefit commonly maintain probiotic use for 14–60 days before outcome assessment, whereas trials reporting insignificance may use only a 5-day course or a low dose (few colony-forming units [CFU]) [129]. A five-day probiotic intervention may be insufficient to detect sustained gastrointestinal or microbiome-mediated benefits. In a systematic review and meta-analysis of 52 randomized controlled trials in irritable bowel syndrome, significant therapeutic effects became apparent after approximately four weeks of supplementation, supporting an intervention period of at least 28 days for comparable clinical endpoints [130,131].

Prebiotics provide substrates that support beneficial microbes or beneficial microbial functions. In dentistry, prebiotics include dietary fibers, arginine, nitrate-rich vegetables, and emerging oral care substrates that promote alkali generation, nitrate reduction, or commensal fitness without feeding acidogenic disease communities. Synbiotics combine probiotics and prebiotics but should be tested as product-specific interventions rather than assumed effective by category [117,121].

Pregnancy and infancy require additional care. Probiotics have been studied in pregnancy, gestational diabetes, infant immune outcomes, eczema risk, and the prevention of necrotizing enterocolitis in selected neonatal populations. Safety is generally favorable in healthy pregnancies, but risk–benefit decisions should be individualized for immunocompromised patients, critically ill infants, and very low-birth-weight newborns [125,126,127,128].

### 13.2. Polyols and Xylitol

Polyols matter because diet shapes oral ecology. Xylitol is nonfermentable or poorly fermentable by mutans streptococci, can reduce acidogenic selection pressure, stimulates salivary flow when delivered as chewing gum or lozenges, and has been shown to reduce mutans streptococci transmission in maternal–child studies [93,94,95,96,132,133,134]. Erythritol has also demonstrated anticaries and antibiofilm effects in experimental and clinical literature. Current evidence supports dose-specific, indication-specific use rather than a one-polyol-fits-all approach. Dental doses are generally well tolerated, but high intake can cause gastrointestinal symptoms, and xylitol is toxic to dogs [133,134,135,136]. The evidence base for xylitol differs sharply by route. In oral health and maternal–child transmission, randomized clinical trials support xylitol, and it is reasonably established in those contexts. Extension to the nasal passage rests on more than mechanism alone. In animal models, xylitol irrigation reduces pneumococcal nasal colonization in rats and enhances bacterial killing in the rabbit maxillary sinus [137,138], consistent with its documented ability to lower the salt concentration of airway surface liquid and unmask innate antimicrobial defenses [139]. Xylitol nasal sprays and irrigations have also demonstrated in vitro and early clinical activity against *Staphylococcus aureus*, *Haemophilus influenzae*, and *Streptococcus pneumoniae*, the same organisms that dominate nasal dysbiosis and feed forward into oral and gastrointestinal pathobiont colonization [139]. Clinical evidence now extends well beyond mechanism: a pilot study and several randomized or prospective trials of xylitol nasal irrigation in chronic rhinosinusitis report modest but consistent improvement in symptom and quality-of-life scores over saline alone, although at least one trial found no additional benefit of xylitol over saline irrigation, and a 2026 systematic review and meta-analysis concluded that xylitol nasal irrigation outperforms saline irrigation in chronic sinusitis on pooled analysis [140,141,142,143,144,145]. In children, a prospective two-center cohort found that xylitol nasal spray reduced recurrent acute otitis media, extending the mother-to-child mutans-transmission logic described above to the nasal compartment and to a different pathogen [146]. A 90-day inhalation toxicology study found no adverse mucosal or systemic effects in rats at xylitol aerosol doses well above intended clinical exposure [147], and reviews of xylitol in otolaryngology practice describe it as a low-risk adjunct across these indications [148]. This nasal literature remains smaller, more heterogeneous in dose and irrigation protocol, and more concentrated in chronic rhinosinusitis than the oral and maternal–child evidence, and mechanistic work connecting xylitol’s antibacterial action to durable, compositional shifts in the nasal microbiome itself, rather than to symptom relief, is still limited [149]. Restoring nasal commensal ecology in parallel with oral microbiome restoration may amplify the benefits of each intervention and reduce the burden of recurrent upper respiratory infections, allergic inflammation, and the persistent airway dysbiosis that predisposes patients to obstructive sleep apnea. The nasal microbiome may, in fact, exert an underappreciated upstream influence on the oral microbiome: a dysbiotic nasal passage continuously inoculates the oropharynx and, through swallowing, the gastrointestinal tract, seeding pathogens and pathobionts into compartments where oral dysbiosis has already compromised colonization resistance.

### 13.3. Remineralization Chemistry and Preserving Commensals

Restoring the oral gateway microbiome also requires mineral and salivary support. Fluoride, hydroxyapatite, arginine, calcium–phosphate systems, and polyphosphates can influence the mineral–biofilm interface. Studies of sodium trimetaphosphate and sodium hexametaphosphate suggest that these agents can act as fluoride-potentiating interfacial modifiers, with nanosized formulations showing promise in enamel demineralization and remineralization models [150,151,152]. These strategies should be integrated with microbial, salivary, and dietary management rather than treated as replacements for ecology-based prevention.

Antimicrobial stewardship is essential. Chlorhexidine, povidone–iodine, essential-oil rinses, antibiotics, and other antimicrobials have appropriate indications. The goal, however, is not chronic sterilization. Long-term indiscriminate suppression can reduce beneficial taxa, alter nitrate reduction, and potentially enrich opportunistic organisms. Dentistry needs the same antimicrobial discipline used in medicine: targeted indication, duration, follow-up, and restoration of microbial homeostasis [48,49,50,51,52]. Table 4 summarizes the ecological intervention framework for homeostasis of the oral gateway microbiome.

## 14. Environmental Exposures, Microplastics, and Materials Science

The oral cavity is also a gateway for environmental particles. Microplastics and nanoplastics can enter through bottled water, food packaging, inhaled dust, consumer products, and potentially dental materials or procedures [153,154,155].Current evidence supports biologic plausibility rather than definitive oral causality. Relevant mechanisms include oxidative stress, NF-κB activation, inflammasome signaling, impaired epithelial repair, macrophage and T-cell polarization, biofilm matrix effects, antibiotic resistance gene transfer, and barrier dysfunction [156,157,158,159,160,161].

This field matters because plastic particles may act as surfaces for microbial biofilms and horizontal gene transfer. Recent reviews propose contamination-controlled measurements of microplastics in saliva, plaque, gingival crevicular fluid, calculus, and tissue; spatial imaging of particles in oral biofilms; and host–microbe co-culture studies to determine whether particles aggravate dysbiosis or periodontal inflammation [156,158]. Inhaled toxicants also link airway exposure to mitochondrial and inflammatory pathways in oral–systemic disease models [159,160]. More research into nasal microplastics is needed; current evidence of the nasal compartment’s microplastic burden is extremely limited, and a dedicated sampling methodology for the sinonasal mucosa has not been standardized. Given that the nasal passage is the primary filter for inhaled particulates and that microplastic particles have already been documented in oral and cardiovascular tissues, the nasal microbiome likely represents a convergence point between inhaled particle exposure and microbial dysbiosis, a relationship that remains almost entirely unexplored [161].

## 15. Evidence Synthesis, Evidence-Level Classification, and Limitations of the Evidence

### 15.1. Evidence-Level Classification of Major Oral–Systemic and Nasal–Systemic Relationships

This narrative review includes heterogeneous evidence due to a lack of consistent research on the relatively new concept of the role of oral and nasal microbiomes in health. Much more information has been published on the role of oral health in systemic health than the nasal gateway (see Table 5).

### 15.2. Limitations of the Evidence and of This Review

This review has several limitations that should inform its interpretation. First, this is a narrative, not a systematic, review; study selection was not exhaustive, was not independently duplicated by a second screener, and is therefore subject to selection and citation bias despite the search parameters described in Section 2. Second, the evidence synthesized here is markedly heterogeneous in design, ranging from mechanistic cell culture and animal model work to large observational cohorts to randomized controlled trials; Table 5 makes this heterogeneity evident. Third, most of the oral–systemic and nasal-systemic relationships discussed are observational or mechanistic; observational association does not establish causation, and confounding by shared risk factors (smoking, diet, socioeconomic status, access to care, comorbid disease) is a plausible alternative explanation for some of the associations reviewed. Fourth, methodological heterogeneity across the underlying microbiome literature, in sampling site, sequencing depth and platform, bioinformatic pipeline, and definition of dysbiosis, limits direct comparison across studies and across the domains reviewed here. Finally, translating any of the relationships in Table 5 into clinical practice, including combined oral–nasal microbiome assessment, requires prospective evidence that such assessment changes diagnosis, management, or outcomes; no such evidence yet exists, and this review’s synthesis should be read as hypothesis-generating for that purpose rather than as clinical guidance.

## 16. Research Agenda

### 16.1. Research Goals

- Define the healthy oral and nasal gateway microbiome by site, age, sex, pregnancy state, diet, airway phenotype, saliva flow, and circadian timing.
- Move beyond bacteria-only assays by including fungi, eukaryotic viruses, bacteriophages, metabolites, extracellular vesicles, and host response markers.
- Use longitudinal designs to determine whether oral or nasal dysbiosis precedes systemic biomarker changes, pregnancy complications, cardiometabolic deterioration, or tumor-associated microbial signatures.
- Validate salivaomics panels with standardized collection protocols, clinically relevant thresholds, reproducibility, and action-linked treatment pathways.
- Test microbiome-restorative interventions as bundled ecology programs that combine biofilm control, diet, airway, sleep, salivary support, probiotics/prebiotics, polyols, and remineralization chemistry.
- Develop maternal–child trials that assess maternal oral microbiome, infant colonization, caries, preterm birth, low birth weight, growth, neurodevelopment, and cardiometabolic risk markers.
- Investigate the origins of the tumor microbiome, including oral sources, intracellular localization, functional genomics, treatment response, and opportunities for targeted microbial modulation.
- Study oral and nasal phage ecology as regulators of bacterial fitness, movement of antimicrobial resistance genes, and precision microbial therapeutics.
- Develop nasal probiotic strategies using strains with documented nasal commensal activity, test delivery routes including nasal spray and nebulization, and evaluate nasal microbiome restoration as a complement to oral microbiome restoration in patients with recurrent upper respiratory infection, allergic rhinitis, and obstructive sleep apnea.
- Investigate the nasal and paranasal sinus microbiome as a modulator of sinonasal nitric oxide production and systemic cardiovascular physiology, with particular attention to whether nasal dysbiosis compounds the cardiovascular risk associated with suppression of the oral nitrate reduction pathway.
- Characterize the nasal and nasopharyngeal microbiome in the context of nasal, nasopharyngeal, or oropharyngeal cancer, and determine whether microbial profiles can contribute to early detection, risk stratification, or treatment response monitoring in populations at elevated cancer risk.

### 16.2. Translational Goals

The clinical endpoint is not sterilization of the mouth or nares. The endpoint is microbial homeostasis, resilient barrier function, salivary competence, airway stability, dietary ecology, early diagnosis, and interprofessional prevention. This is the practical meaning of the oral–nasal gateway microbiome. Achieving that endpoint will require cooperation that currently does not exist at a structural level. Otolaryngologists and dentists manage adjacent and functionally interdependent ecosystems, yet they rarely coordinate care for shared patients. A patient with chronic rhinosinusitis and periodontal disease is experiencing dysbiosis at both ends of an anatomically connected mucosal system; treating one without the other is biologically incomplete. ENT and dental professionals must develop shared screening frameworks, referral pathways, and co-management protocols that reflect the nasal–oral–systemic continuum described in this review. As the evidence summarized in Section 15 matures, a reasonable long-term goal is for patients to receive periodic airway screening and oral microbiome risk assessment, analogous to blood pressure and glucose measurement; consistent with Section 9.3 and Section 15.2, this is a translational goal rather than a current standard of care, since no prospective evidence yet shows that combined assessment changes diagnosis, management, or outcomes. Delayed recognition of untreated OSA, unchecked oral dysbiosis, nasal pathobiont seeding of the gastrointestinal tract, and progressive systemic inflammatory burden may plausibly be mitigated by earlier, interprofessional attention, although this hypothesis has not been tested prospectively. Validated, standardized salivary diagnostics could enable earlier detection of airway microbiome shifts, systemic disease trajectories, and the nasal–oral–gut pathobiont burden that currently goes undetected until structural damage has occurred. Integrating salivaomics into dental and airway visits, paired with nasal microbiome assessment and xylitol-based nasal and oral prebiotic strategies, is a translational research priority for biologically informed, interdisciplinary prevention, pending the prospective validation called for in Section 15.2.

## 17. Conclusions

Current evidence, summarized by evidence level in Table 5, supports viewing the oral microbiome as a gateway influencing systemic physiology and distal microbial ecosystems, while the strength of that evidence varies considerably across the clinical domains reviewed and is, at present, weaker for the nasal component of the model than for the oral component. It is a specialized, co-evolved airway–digestive ecosystem that protects the host when balanced and contributes to oral and systemic disease when dysbiotic. Its gateway status is supported by anatomy, continuous salivary flow and swallowing, vascular exposure through inflamed periodontal surfaces, oral–gut microbial transfer, oral–airway interactions, nitrate–nitrite–nitric oxide physiology, maternal–child microbial transmission, and the appearance of oral organisms in distal diseases and tumor ecosystems.

Bacteria, fungi, viruses, and bacteriophages all belong in this model. A comprehensive oral–systemic model must include commensal protection, cross-kingdom biofilms, phage-mediated microbial evolution, human viral oncogenesis and immune modulation, salivary biomarkers, host response signaling, and environmental exposures. Oral health can no longer be reduced to teeth, gingiva, plaque removal, or restorative repair.

Salivaomics should provide a practical diagnostic platform for translating this biology, although the number of salivary biomarkers already cataloged across human diseases reflects the scale of the research opportunity rather than present clinical readiness; each candidate panel still requires independent analytical validation, clinical validation, and demonstrated interpretability before diagnostic deployment. With that caveat, saliva may in time provide an accessible, repeatable, noninvasive window into microbial, inflammatory, metabolic, oncologic, and neurodegenerative risk. When combined with validated microbiome testing, airway evaluation, maternal and child prevention strategies, probiotics, prebiotics, polyols, remineralization chemistry, and antimicrobial stewardship, salivaomics can support a more preventive and biologically integrated model of dental practice.

## Figures and Tables

**Table 1:** Components of the oral gateway microbiome and representative roles.

Component	Representative Taxa	Beneficial Roles	Dysbiotic/Pathogenic Roles
Bacteria	Streptococcus, Veillonella, Neisseria, Rothia, Actinomyces, Fusobacterium, Prevotella, Porphyromonas, Treponema, Tannerella, Aggregatibacter	Colonization resistance, pH buffering, early biofilm structure, nitrate reduction, and mucosal immune education	Caries, gingivitis, periodontitis, peri-implantitis, bacteremia, oral–gut translocation, inflammatory burden
Fungi	*Candida albicans*, *Candida dubliniensis*, *Candida tropicalis*, Malassezia, Saccharomyces, and other low-abundance fungi	Low-biomass commensalism, mucosal immune training, cross-kingdom signaling	Candidiasis, denture stomatitis, severe early childhood caries consortia, mucosal inflammation, cancer-associated shifts
Human viruses	HSV-1, HSV-2, cytomegalovirus, Epstein–Barr virus, papillomaviruses, and other eukaryotic viruses	Immune surveillance and latent virus–host interactions	Mucosal ulceration, periodontal immune modulation, oncogenesis in HPV-associated oropharyngeal cancer, and immunosuppression-associated disease
Bacteriophages	Streptococcal, Actinomyces, Fusobacterium, and other oral phages	Population control, microbial diversification, biofilm regulation, and CRISPR-phage dynamics	Potential virulence-gene movement, antimicrobial resistance gene transfer, altered bacterial fitness, and dysbiosis
Host-saliva matrix	Mucins, IgA, antimicrobial peptides, enzymes, proteins, metabolites, electrolytes, extracellular vesicles	Lubrication, digestion, remineralization, microbial containment, and biomarker reservoir	Xerostomia, impaired buffering, altered pellicle, increased dysbiosis, and diagnostic signals

**Table 2:** Evidence-supported routes linking the oral gateway microbiome to systemic biology.

Route	Mechanism	Representative Organisms/Mediators	Clinical Domains
Swallowing/oral–gut axis	Continuous ingestion of saliva and biofilm organisms; survival of selected taxa; gut colonization under permissive conditions	*P. gingivalis*, *F. nucleatum*, Streptococcus, Veillonella; LPS; short-chain and amino acid metabolites	IBD, liver disease, colorectal cancer, metabolic inflammation
Barrier breach and bacteremia	Inflamed gingiva and periodontal pockets permit microbial products and transient bloodstream exposure	*P. gingivalis*, *A. actinomycetemcomitans*, *F. nucleatum*, *C. rectus*; endotoxin; CRP; IL-1β, IL-6, TNF-α	Atherosclerosis, diabetes, pregnancy complications, and endocarditis risk contexts
Immune–metabolic signaling	Local dysbiosis sustains innate and adaptive immune signaling and alters insulin and endothelial pathways	Gingipains, complement crosstalk, inflammasome activation, and neutrophil priming	Diabetes, obesity, NAFLD/NASH, rheumatoid arthritis, neuroinflammation
Nitric oxide biology	Oral nitrate-reducing taxa generate nitrite from dietary nitrate, affecting nitric oxide bioavailability	Neisseria, Rothia, and nitrate-reducing consortia	Blood pressure, endothelial function, exercise response
Airway aspiration and dryness	Oral pathogens can be aspirated; mouth breathing dries the mucosa and reshapes biofilms	Respiratory pathogens, anaerobic oral taxa, and xerostomic biofilms	Pneumonia risk, COPD, OSA–periodontitis overlap
Microbial and host extracellular vesicles	EVs carry microbial products, RNA, proteins, metabolites, and inflammatory signals across barriers	Maternal microbiota-derived EVs; bacterial OMVs; host exosomes	Pregnancy immune priming, inflammation, and cancer signaling

**Table 3:** Salivaomics domains and clinical relevance.

Salivaomics Domain	Examples of Analytes	Current or Near-Term Clinical Relevance
Microbiomics	Bacterial, fungal, viral, and phage profiles; pathogen panels and dysbiosis indices	Caries, periodontal and peri-implant risk; oral–systemic risk stratification; monitoring treatment response
Proteomics	Cytokines, immunoglobulins, enzymes, matrix metalloproteinases, and antimicrobial peptides	Inflammation, tissue breakdown, immune status, periodontal activity, and oral cancer research
Transcriptomics	microRNA, mRNA, circular RNA, and long noncoding RNA	Cancer, neurodegeneration, inflammatory disease, and treatment response research
Genomics/epigenomics	Host DNA, cell-free DNA, methylation, telomere-related measures, and circulating tumor DNA signals	Precision risk detection, cancer screening research, and host susceptibility
Metabolomics/lipidomics	Amino acids, short-chain fatty acids, lipids, lactate, nitrate/nitrite, oxidative-stress products	Diet-microbiome function, metabolic disease, inflammatory activity, nitric oxide biology
Extracellular vesicles	Host exosomes and microbial vesicles carrying proteins, RNA, and metabolites	Maternal-fetal signaling, cancer biology, and inflammatory monitoring

**Table 4:** Ecological intervention framework for oral gateway microbiome.

Intervention Domain	Primary Target	Examples	Cautions
Mechanical biofilm control	Biofilm mass and inflammatory load	Brushing, interdental cleaning, periodontal maintenance, peri-implant monitoring	Technique and adherence determine outcome; structural care alone cannot correct diet or airway drivers
Diet and prebiotics	Substrate selection and microbial metabolism	Reduced frequency of sugar intake, fiber, arginine, nitrate-rich vegetables, and hydration	Avoid feeding acidogenic communities; individualize for medical conditions
Airway and sleep	Dryness, oxygen tension, hypoxia, inflammation	Nasal breathing support, OSA screening, orthodontic/ENT collaboration, smoking/vaping cessation	Dental sleep appliances, when medically indicated and coordinated with OSA care
Probiotics/synbiotics	Colonization resistance and immune modulation	*L. reuteri*, *L. rhamnosus*, *L. plantarum*, *L. paracasei*, *Bifidobacterium* spp., *S. salivarius* K12/M18	Effects are strain- and indication-specific
Polyols	Acidogenic selection and pathogen growth phenotypes	Xylitol gum/lozenges; emerging polyol research	Dose matters; gastrointestinal tolerance
Remineralization chemistry	Mineral–biofilm interface	Fluoride, hydroxyapatite, arginine, TMP/HMP, calcium-phosphate systems	Should be paired with microbial ecology and salivary support
Antimicrobial therapy	Pathogen burden during active disease	Chlorhexidine, local antimicrobials, and antibiotics when indicated	Avoid chronic indiscriminate suppression; preserve beneficial commensals

**Table 5:** Evidence-level classification of major oral–systemic and nasal–systemic relationships discussed in this review.

Oral–Systemic Relationship	Representative Evidence	Evidence-Level Classification	Key References
Periodontal disease ↔ type 2 diabetes (bidirectional)	Multiple RCTs of periodontal therapy show modest HbA1c reduction; large observational cohorts show bidirectional association.	Supported by clinical intervention studies	[20,21,22,23,24,25,26,27,28]
Periodontal pathogens ↔ atherosclerotic cardiovascular disease	Consistent observational association; mechanistic/experimental evidence for bacteremia and vascular invasion; no intervention trial has shown periodontal therapy prevents cardiovascular events.	Associated in observational studies; demonstrated in experimental models; published case–control studies	[20,21,22,23,24,25,26,27,28]
Maternal periodontal disease/*F. nucleatum* → adverse pregnancy outcomes	Strong observational association and animal model mechanistic data; periodontal treatment intervention trials have not reduced preterm birth.	Associated in observational studies; not yet established clinically as a preventable outcome; periodontal treatment trials criticized as more causative than preventive	[38,39,40,41,42,43,44,89,90,91,92]
Oral pathobiont translocation → gut dysbiosis/IBD	Ectopic gut colonization by oral taxa demonstrated in gnotobiotic mouse models; observational enrichment in IBD cohorts.	Demonstrated in experimental models; associated in observational studies	[9,20,21,22,23,24]
Fusobacterium nucleatum → colorectal cancer progression	Mechanistic and xenograft evidence for tumor persistence and growth promotion; consistent tumor tissue enrichment in observational series; causation in human carcinogenesis unproven.	Demonstrated in experimental models; associated in observational studies	[97,98,99]
Oral/pancreatic microbiome → pancreatic cancer	Mouse model evidence for immune-suppressive oncogenesis; observational microbial signatures in resected tumors; independent replication and methodological disputes ongoing for cancer microbiome signals generally.	Demonstrated in experimental models; associated in observational studies; not yet established clinically	[97,98,99,100,101,102,103,104,105]
*P. gingivalis* → Alzheimer’s disease/neurodegeneration	Mechanistic gingipain/neuroinflammatory data and observational association.	Demonstrated in experimental models; associated in observational studies; not established clinically	[85,86,87,88]
Periodontal disease ↔ rheumatoid arthritis	Observational association and citrullination pathway mechanistic plausibility; no intervention trial demonstrates periodontal treatment alters RA course.	Associated in observational studies; mechanistically plausible; not yet established clinically	[85,86,87,88]
Nasal microbiome dysbiosis ↔ obstructive sleep apnea	Cross-sectional correlations only; directionality (cause, consequence, correlate, or modifier) is explicitly unresolved.	Associated in observational studies only; not yet established clinically	[8,37,50,51,52,53]
Xylitol → oral/maternal–child mutans streptococci reduction	Multiple randomized controlled trials in maternal–child cohorts and general populations.	Supported by clinical intervention studies	[93,94,95,96,132,133,134]
Xylitol nasal sprays/irrigation → nasal dysbiosis reduction	Animal and in vitro antibacterial data; a pilot study, several randomized/prospective irrigation trials, and a 2026 meta-analysis in chronic rhinosinusitis; one pediatric otitis media-prevention cohort; nasal microbiome-specific compositional data still lacking.	Supported by clinical intervention studies in chronic rhinosinusitis; preliminary in pediatric otitis media prevention; not yet demonstrated as nasal microbiome restoration	[137,138,139,140,141,142,143,144,145,146,147,148,149]
Oral probiotics → caries/periodontal disease modulation	Multiple randomized trials with strain-specific and duration-dependent effects; results heterogeneous across strains and outcomes.	Supported by clinical intervention studies (strain- and protocol-dependent)	[116,117,118,119,120,121,122,123,124,125]
Nasal probiotics → airway/allergic disease modulation	Mechanistic rationale by analogy to gut/oral probiotics; no dedicated nasal probiotic efficacy or safety trials identified.	Not yet established clinically (hypothesis-generating)	[126,127]
Salivary biomarker panels → caries/periodontal risk detection	Analytically validated assays in clinical use or advanced clinical validation.	Supported by clinical intervention/validation studies	[106,107,108,109,110,111,112,113,114,115]
Salivary biomarker panels → cancer, cardiovascular, neurodegenerative, or airway dysbiosis risk detection	Biomarker cataloging and discovery-stage studies; analytical and clinical validation not yet established for routine use.	Not yet established clinically (research stage) but clinical trials ongoing	[106,110]

## Data Availability

No new data were created or analyzed in this review. Data sharing is not applicable to this article.
